# Change in adverse event reporting following immunization of hepatitis B vaccine among infants between 2013 to 2020 before and after the vaccine administration law in China

**DOI:** 10.3389/fimmu.2022.956473

**Published:** 2022-09-30

**Authors:** Chao Wang, Ninghua Huang, Qing-Bin Lu, Steven Black, Xiaofeng Liang, Fuqiang Cui

**Affiliations:** ^1^ Department of Laboratorial Science and Technology and Vaccine Research Center, School of Public Health, Peking University, Beijing, China; ^2^ Global Center for Infectious Disease and Policy Research, Peking University, Beijing, China; ^3^ Global Center for Infectious Disease and Policy Research and Global Health and Infectious Diseases Group, Peking University, Beijing, China; ^4^ Department of Pediatrics, Cincinnati Children’s Hospital, Cincinnati, OH, United States; ^5^ Institute of Disease Control and Prevention, Jinan University, Guangzhou, China

**Keywords:** hepatitis B vaccine, safety, adverse events following immunization, infants, vaccine administration law

## Abstract

**Background:**

Hepatitis B vaccine (HepB) has been routinely recommended as part of the immunization program in China and has had a satisfactory safety and effectiveness profile in protecting infants from hepatitis B virus infection. We evaluated the surveillance sensitivity and changes over time of AEFI reports related to HepB among infants based on the consistent national data before and after the introduction of vaccine administration law (LAW) from 2013 to 2020 in China.

**Methods:**

AEFI records were extracted from the Chinese National AEFI Surveillance System from 2013 to 2020. According to the proportion of different kinds of HepB vaccines distributed, the annual administration data of the most distributed HepB produced by Bio-Kangtai and its corresponding adverse reaction reports were collected and analyzed. We categorized the time interval into the pre-LAW period (2013 to 2017), transition period (2018 to 2019), and LAW period (2020) to demonstrate the impact of LAW on the surveillance patterns of AEFIs.

**Results:**

The annual AEFI rates increased from 3.1/100,000 to 14.8/100,000 over this period in total. The rate ratio for the post-LAW period and pre-LAW period was 2.19 (95%CI: 2.10, 2.29). Common reactions occupied 87.6% of the total reported AEFIs whose rate was recorded as 7.9/100,000. Rare reactions occupied 9.1% of the total AEFIs showing an average rate of 0.8/100,000, of which anaphylaxis accounted for over 80%, with the rate ratio of the transition period and LAW period as 1.36 (95%CI:1.22, 1.52) and 1.14 (95%CI:0.95, 1.35), respectively. Children receiving more than one vaccine showed a higher proportion of fever, anaphylaxis, and febrile convulsions, which were suggested to be a result of vaccine co-administration vaccines, such as the DPT and Polio vaccine.

**Conclusion:**

Most reactions were mild and self-limited and the rates of rare more serious events remained stable. The LAW has largely increased the surveillance capability and sensitivity on AEFIs of HepB and also contributes to enhancing public confidence in HepB immunization. Hepatitis B vaccination is a safe and effective means of preventing the complications of hepatitis B disease and continuous standardized AEFI investigation and assessment of causal association should be maintained.

## Highlights

- It is important as high vaccine uptake is critical to preventing infection which has a heavy disease burden on Chinese society and public health. With the increase in surveillance sensitivity, the pattern of changes in AEFI reporting has not been explored to date.
- The vaccine administration law largely increased the sensitivity of AEFI surveillance following HepB immunization.
- Among the most common AEFIs reported, the vast majority were local and systemic reactions including fever, redness, and induration.
- This study demonstrated that the number and characteristics of severe abnormal reactions has remained stable.
- The sensitivity of AEFI surveillance improved following HepB immunization events from 2013 to 2020, and the AEFIs associated with HepB among children below 12 months remained predictable, reaffirming its satisfied safety in utilization.
- The LAW has largely increased the surveillance capability and sensitivity on AEFIs of HepB and also contributes to enhancing public confidence in HepB immunization.

## Background

The Hepatitis B vaccine (HepB) is the most economical and effective means of preventing hepatitis B virus infection (1). HepB was introduced into an expanded program on immunization management in 1992 and fully integrated as a free infant vaccine in the national China immunization program in 2002, given at birth, one, and six months respectively, and has generally demonstrated a satisfactory safety and effectiveness in China (2, 3).

For vaccines in general, as vaccine-related diseases are well controlled, concern regarding adverse events following immunization (AEFI) increases (4, 5). China developed its AEFI surveillance system in 2005 and implemented it nationwide in 2010 (6). Continuous passive surveillance monitoring of vaccine safety to identify AEFI is very important for, detecting new adverse events following immunization and classification of vaccine-related AEFIs and coincident issues, thus improving the quality and the utilization of vaccines (7).

The coverage of HepB among newborn infants has now reached over 99% after falling to 60% after the Bio-Kangtai HepB issue in 2013 (8, 9). Vaccine practitioners indicate that the prolonged damage to the public’s confidence in HepB caused by this issue remains a potential time bomb that could lead to a future drop in coverage, even though AEFI has been only rarely reported for HepB nationally (8, 9).

Considering the differences in monitoring capabilities and completeness for AEFI reporting in China over time, the description of dynamics of reporting of AEFIs by specific HepB vaccine and by year is of great significance for further improving the monitoring system and enhancing the confidence of the Chinese population (10, 11).

In 1992, Merck Vaccines transferred the technology for their newly US-licensed yeast-derived recombinant hepatitis B vaccine (HepB) to Chinese manufacturer Bio-Kangtai for production and use in China. That year, China implemented universal HepB vaccination of infants with BKT HepB. In recent years, China has experienced some vaccine events with great impact, including the HepB issue which was related to a vaccine produced by Bio-Kangtai biological company. From December 2013 to January 2014, eighteen infants’ deaths after Bio-Kangtai Hep-B immunization were intensively and widely reported through public media. Before they were clarified as coincidence events, Bio-Kangtai Hep-B was suspended by the Chinese Food and Drug Administration for more than one month. Suspicion and worries about its safety caused by such an issue among parents then resulted in a more than 30% decrease in coverage of HepB among newborn infants from 2013 to 2014 (9, 12–14).

Various vaccine events similar to the Bio-Kangtai Hep-B issue in recent years have opened the window for vaccine-related policy, including the Chinese vaccine administration law (LAW). The draft of LAW was then announced in 2018 and discussed in the “two sessions” of China in 2019 before it entered into force at the end of 2019. The main aspects including vaccine development and registration, vaccine production and batch issuance, vaccine circulation, abnormal reaction monitoring and treatment, post-marketing management of vaccines, et al. were widely discussed across the society and stipulated in the LAW. Such a process has been believed as an in-time opportunity to improve consciousness of the importance of vaccination as well as AEFIs among all the interested parties. It allows for more standardized evaluation and management of vaccination in China. However, there have been rare reports on whether LAW’s introduction increased the surveillance sensitivity on AEFIs.

In this study, we evaluated the AEFIs related to HepB produced by Bio-Kangtai among the children aged below 12 months old, by different characteristics of age, gender, and the period concerning the implementation of LAW, based on the consistent national data of the target-specific HepB vaccine from 2013 to 2020 in China. We aimed to provide evidence to support the public perception of the safety of this vaccine, offer sensitivity analysis of the passive surveillance of AEFIs before and after the LAW, and hence increase public confidence in HepB vaccination.

## Methods

### The National AEFI Surveillance System (NASS)

NASS is a passive surveillance system that was established in 2005 and is operated collaboratively by the Chinese Center for Disease Control and Prevention (CDC) and the Chinese Food and Drug Administration (http://219.141.175.204/) (6, 15), based on *Chinese Regulations on the Administration of Vaccine Circulation and Vaccination* and *Work Standard on Vaccination* issued in 2005 (both updated in 2016). Following the AEFI surveillance guidelines of the World Health Organization and Chinese National Health Commission as *Identification method for Adverse Reaction following Vaccination* in 2008 and *National Surveillance Guideline for Adverse Reaction following Immunization* in 2010 (6, 16), the surveillance on AEFI in China had been largely improved by making clear the responsibilities of every interested party in NASS. Reports of AEFIs are submitted to the report system by vaccine providers, practitioners, CDC, and health care institution. Each reported AEFI should be investigated by the national or municipal CDC. Reports of AEFI should contain the following information in a standardized fashion: date of report, age and sex of patient, kind and lot of suspect vaccine(s), description of the AEFI, time interval after immunization, the final diagnosis of AEFI, and any other additional remarks from the reporter (6, 11, 16). The above issues have been guaranteed by the LAW while they were suggested not well implemented across China previous to the LAW (16).

### Data collection

The database provided by the NASS is subdivided into vaccine categories, which allows us to extract the targeted reports on AEFIs of HepB. For this study, cases of children below 12 months old were evaluated. AEFI refers to the reactions unrelated to the purpose of vaccination or unexpected reactions caused by the characteristics of the vaccine itself, which are related to the individual differences of the recipients, including common reactions and rare reactions. We searched for patterns in AEFIs according to coding terms, clinical diagnosis, anamnesis, vaccination date, gender, age, time to onset, and administration of a single vaccine versus multiple vaccines. Reports on AEFIs of HepB had been suggested to experience increasing surveillance sensitivity since the Bio-Kangtai HepB issue happened in 2013. Then, special attention was paid to reports of serious events from 2013 to as late as 2020 due to the postponement of data release from NASS. The annual number of administered doses of HepB in mainland of China during the study interval of 2013 to 2020 was obtained from the online batch issuance system of China which then can be used to provide an estimation of reporting rates on AEFIs following HepB vaccination. According to the proportion of different kinds of HepB administered, the annual issuance data of HepB produced by Bio-Kangtai and its corresponding adverse reaction reports were collected and analyzed.

### Assessment of severity and reactions

All AEFIs were assessed as non-serious or serious and further subdivided into the following categories of severity including the definition for “serious” AEFI proposed by the WHO: non-serious (mild severity), with no intervention necessary; non-serious (moderate severity), with medication given or physician visit or event interfering with daily activities or loss of working hours; and serious (severe), with any untoward medical occurrence that results in death, hospitalization or prolongation of hospitalization, or persistent or significant disability/incapacity or is life-threatening. Each AEFI record lists several symptoms, signs, and/or diagnoses that have been recorded by municipal and/or county level CDC staff from the reporter’s description into standardized terms according to guidelines for the identification of AEFI issued by the MoH of China in 2008. Common reaction: the reaction that occurs after vaccination and is caused by the inherent characteristics of the vaccine itself, which will only cause transient physiological dysfunction to the body, mainly including fever (classified into three levels: < 37.0℃, 37.1℃ to 38.5℃, ≥ 38.6℃), local redness (classified into three levels: none, 0.1cm to 2.5cm, ≥ 2.6cm), swelling, and induration (classified into three levels: none, 0.1cm to 2.5cm, ≥ 2.6cm) in this study. Rare reaction: the qualified vaccine causes damage to the tissues, organs, and functions of the recipient during or after the implementation of the standard vaccination, and the relevant parties are not at fault, mainly including. Anaphylactic reaction, Nervous system reaction, injection site reaction, and other reactions in this study. A summary of the vaccine reactions by severity level was listed in **Table S1**. The national immunization program for child immunization procedures was listed in **Table S2**.

### Data analysis

Reported cases and rates of AEFI were calculated and stratified by clinical diagnosis and disease categories as well as different characteristics including age, vaccination onset interval, and seriousness. Co-administration of one or more vaccines besides HepB was categorized in reporting the disease categories. Considering the process of LAW in China, we categorized the time interval into the pre-LAW period (Period I: 2013 to 2017), transition period (Period II: 2018 to 2019), and LAW period (Period III: 2020). To demonstrate the impact of LAW on the surveillance patterns of AEFIs, average incidence rates of AEFI of the three separate LAW stages were calculated. The average incidence rates of AEFI among three LAW periods were then compared by using rate difference (RD)and rate ratio (RR)with the Wilson method, as well as their 95% confidence interval (95% CI).

## Results

### Summary of AEFI reports, 2013-2020

**Figure 1:**
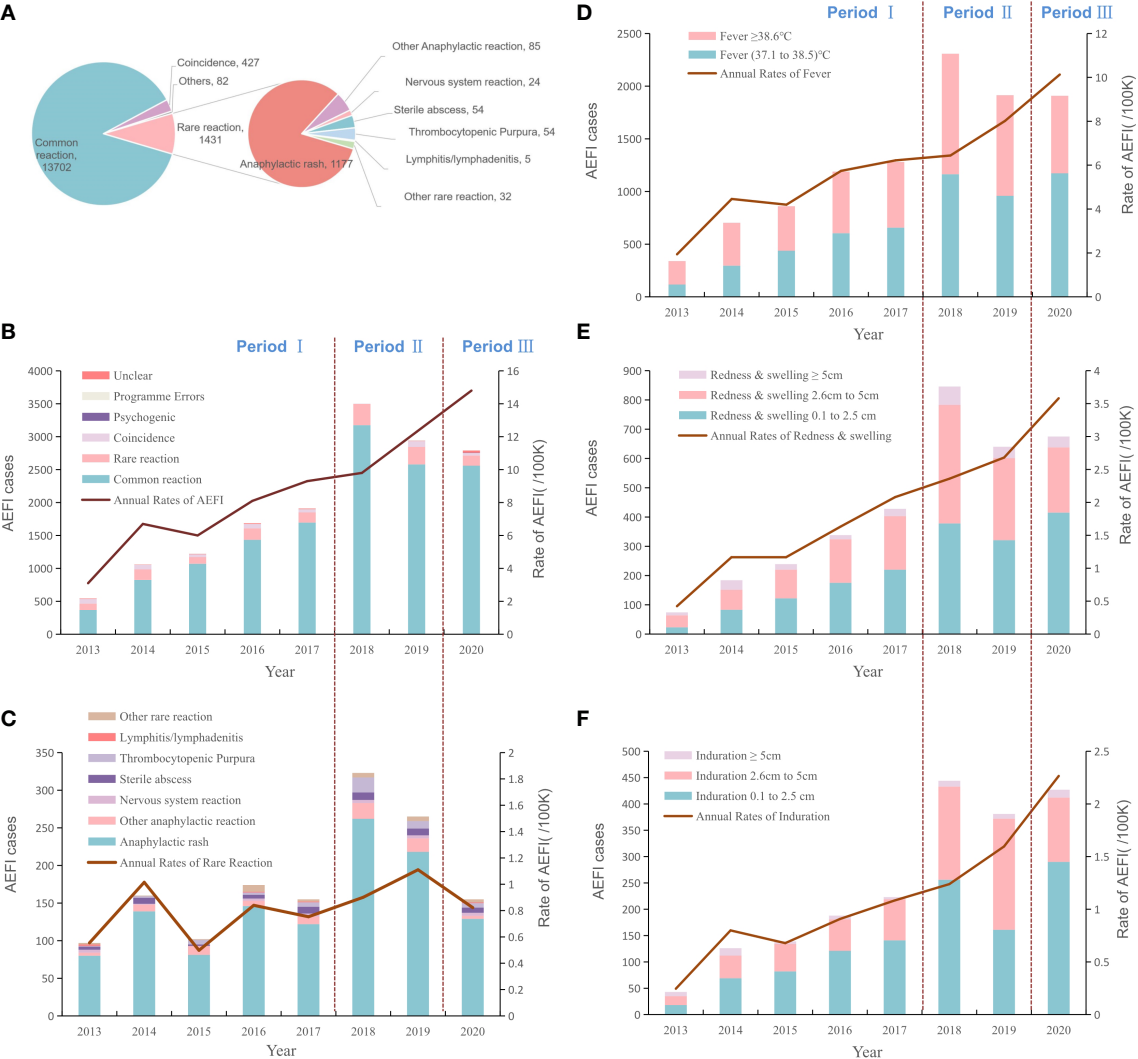
The proportion of reported AE cases and rates of adverse events after HepB immunization by years and reaction categories. **(A)**. The proportion of total AEFIs after HepB immunization and the composition of the rare reactions **(B)**. The dynamic changes of total rates and proportions of AEFIs following HepB by year **(C)**. The dynamic changes of rates and proportions of rare reactions following HepB by year **(D)**. The dynamic changes of rates and proportions of fever following HepB by year **(E)**. The dynamic changes of rates and proportions of redness & swelling following HepB by year **(F)**. The dynamic changes of rates and proportions of induration cases following HepB by year.

From January 2013 to December 2020, NASS received 15,462 HepB-associated AEFIs among infants. The recorded annual administered doses of 10μg BIOKANGTAI HepB vaccination ranged from 15760846 to 35837330 between 2013 and 2020. The annual AEFI rates increased from 3.1/100,000 to 14.8/100,000. The majority of AEFI (97.7%, 15290/15462) were reported among children aged between 30 to 365 days that had received 10μg HepB vaccination. And 55.4% (8668/15462) were males, 94.0% of AEFIs appeared within 3 days after injection; serious AEFI cases occurred at a rate of 3.3% of the total AEFI cases (**Tables 1**, **2**). A spike was found in the recorded amount of AEFI cases in 2018, and a subsequent decrease from 2019 to 2020, while a dramatic increase was found in 2018 and kept an increasing trend during 2019 and 2020 as 12.3/100,000 and 14.8/100,000, respectively. Common reactions occupied 87.6% of the total reported AEFIs whose rate was 7.9/100,000. Rare reactions occupied 9.1% of the total AEFIs showing an average rate of 0.8/100,000 (**Figure 1**, **Table 1**).

**Table 1:** Rates of AEs after Hep-B vaccination by years and reaction category.

		Cases	Proportion of the total cases	Administrated doses	Rates (/100,000 vaccinated doses)
Years	2013	544	3.5	17475606	3.1
	2014	1055	6.7	15760846	6.7
	2015	1220	7.8	20477238	6.0
	2016	1685	10.8	20711183	8.1
	2017	1909	12.2	20577428	9.3
	2018	3499	22.4	35837330	9.8
	2019	2940	18.8	23868086	12.3
	2020	2790	17.8	18848042	14.8
Reaction category	Common reaction	13702	87.6	173555759	7.9
	Rare reaction	1431	9.1	173555759	0.8
	Coincidence	427	2.7	173555759	0.2
	Psychogenic	2	<0.1	173555759	<0.1
	Programme Errors	2	<0.1	173555759	<0.1
	Unclear	78	0.5	173555759	<0.1
					
Total		15642	100	173555759	9.0

**Table 2:** Basic characteristics of the AEFI cases from NASS.

Characteristics		Total Cases		Male			Female	
		n	%	n		%	n	%
Age group	Within 24 hours	206	1.3	120	58.3		86	41.7
	25 to 72 hours	73	0.5	41	56.2		32	43.8
	4 to 14 days	18	0.1	10	55.6		8	44.4
	15 to 29 days	55	0.4	29	52.7		26	47.3
	30 to 365 days	15290	97.7	8468	55.4		6822	44.6
Onset interval	Within 1 day	7334	46.9	4066	55.4		3268	44.6
	1 to 2 days	6147	39.3	3376	54.9		2771	45.1
	2 to 3 days	1228	7.8	696	56.7		532	43.3
	≥3 days	933	6.0	530	56.8		403	43.2
Seriousness	Non-serious AEFI	15130	96.7	8356	55.2		6774	44.8
	serious AEFI	512	3.3	312	60.9		200	39.1
Co-administration	None	7946	50.8	4344	54.7		3602	45.3
	1 more vaccine	7350	47.0	4125	56.1		3225	43.9
	DPT	1223	16.6	699	57.2		524	42.8
	Polio	824	11.2	465	56.4		359	43.6
	MenA/C	4456	60.6	2460	55.2		1996	44.8
	MMR	366	5	227	62.0		139	38.0
	Others	481	6.5	274	57.0		207	43.0
	2 more vaccines	346	2.2	199	57.5		147	42.5
	Polio + DPT	183	52.9	106	57.9		77	42.1
	Polio + MenA/C	111	32.1	65	58.6		46	41.4
	Polio + MMR	12	3.5	6	50.0		6	50.0
	MenA/C +ROTA	20	5.8	8	40.0		12	60.0
	Others	20	5.8	14	70.0		6	30.0
Total		15642	100	8668	55.4		6974	44.6

DPT, Diphtheria, pertussis and tetanus mixed vaccine; Polio, Polio vaccine; MenA/C, Meningococcal A/C vaccine; MMR, Measles, mumps, rubella vaccine; ROTA, Rotavirus vaccine.

### AEFI of HepB with co-administered vaccines

We compared the proportions of AEFI cases that received HepB alone or with other vaccines. Forty-seven percent (7350/15462) of AE cases were reported following co-administration of one more vaccine, which is far more than that of two more vaccines. Group A+ C meningococcal polysaccharide vaccine (MenA/C vaccine) was reported as the most frequent co-vaccine of AEFI cases with 60.6% (4456/7356) records. Two more vaccines co-administrated showed much fewer AEFI reports with 346 out of 7350 (4.7%) cases in total. And males showed more AEFIs compared to female infants in general. Further, we found that co-administrated cases showed a higher proportion of fever, anaphylaxis shock, and febrile convulsion (**Table 2**, **Table S3**). Of the four anaphylaxis shock cases with co-administered vaccines, one was co-administrated with pertussis, diphtheria, and tetanus mixed (DPT) vaccine, one was with Meningococcal vaccine, and two with the polio vaccine. Of the 12 febrile convulsion co-administrated cases, seven were co-administrated with the Meningococcal vaccine, four were with the DPT vaccine, and one was with the polio vaccine. Further, we found a case of acute paralysis syndrome reported by a two-year-old girl, who had received co-administrated HepB and oral polio vaccine.

### Common AEFIs associated with hepatitis B vaccine

Of 35.0% (5407/15462) cases were reported fever ranging between 37.1°C and 38.5°C, with the annual rates from 0.7/100,000 to 6.2/100,000. Cases with a fever over 38.5°C accounted for 33.0% (5095/15462) of the total AEFIs we collected, and the average rate was 2.9/100,000. Reported redness and swelling of 0.1cm~2.5cm, 2.6cm~5cm or over 5cm were 50.7% (1737/3424), 42.3% (1447/3424) or 0.7% (240/3424), and the average rates were 1.0/100,000, 0.8/100,000 or 0.1/100,000, respectively. The rates of induration after immunization were 0.7/100,000 for 0.1cm~2.5cm, 0.4/100,000 for 2.6cm~5cm, or <0.1/100,000 for 5cm and over (**Figure 1**, **Table S4**).

### Rare AEFIs following HepB vaccination

The average rare reaction rate was estimated at 0.8/100,000. The majority of rare AEFIs (92.0%, 1316/1431) were anaphylactic reactions. The most frequent rare reaction was recorded as anaphylactic rash, followed by sterile abscess and thrombocytopenic (purpura), with rates of 6.8/million, 0.3/million, or 0.3/million, respectively. The average rates of angioedema, allergic purpura, anaphylaxis shock, and Arthus reaction were around 0.1/million. In contrast to the rates of common reactions, the annual rates of rare reactions from 2013 to 2020 remained stable 2013 to 2020 which was generally around 0.8/100,000 vaccinees (**Figure 1** and **Table S5**).

### Death after Hep-B immunization

During the 2013 to 2020 period, 132 (0.8%, 132/15642) deaths after Hep-B vaccination were reported, among which 97.7% (129/132) death cases were unrelated to vaccination as they were due to other medical conditions. One death was followed by a program error that the infant was administrated a co-vaccine of Bacille Calmette-Guérin vaccine (BCG) outside the recommended age range, and died of food asphyxia with an allergic reaction. Two deaths were with diagnosed rare reactions after vaccination, which were recorded in 2017 and 2020. These two deaths were both males; one was co-administrated DPT vaccine and died of Primary endocardial least fibrosis; the other one was co-administrated polio and died of epilepsy & intracranial hemorrhage.

### Trends of the reported rates of AEFIs between different LAW periods

The Average rate of total AEFIs during the pre-LAW stage was 6.75 per 100 thousand doses, which was dramatically lower than that during the LAW period at 14.8 per 100 thousand doses, with the RR as 2.19 (95%CI: 2.10, 2.29). Rates of common reaction of the separated three time intervals (pre-LAW, transition, and LAW) were 5.67, 9.64, or 13.58 cases per 100 thousand doses respectively. RRs for transition period and LAW period were 1.70 (95%CI: 1.64, 1.76) or 2.39 (95%CI: 2.28, 2.51). And rate changes for fever, redness, or induration showed similar patterns in which the largest RR was found for the induration rate in the LAW period as 2.99 (95%CI: 2.66, 3.37). In contrast, the rate differences and rate ratios were found to be much less for rare reactions, at 1.36 (95%CI:1.22, 1.52) for RD or 1.14 (95%CI:0.95, 1.35) for RR of LAW period compared to pre-LAW period (**Table 3**, **Figure 1**).

**Table 3:** Average rate of AEs following HepB of different stages of LAW conduction from 2013 to 2020 (per 100 thousand doses).

Adverse events		Average Rates (/100K vaccinated doses)	Rate difference (/100K vaccinated doses)	Rate ratio
Total AEFI	Pre-LAW	6.75 ± 0.06	Ref.	Ref.
	Transition period	10.78 ± 0.16	4.03 (3.75, 4.33)	1.60 (1.54, 1.65)
	LAW	14.80 ± 0.64	8.05 (7.53, 8.09)	2.19 (2.10, 2.29)
Common reaction	Pre-LAW	5.67 ± 0.06	Ref.	Ref.
	Transition period	9.64 ± 0.15	3.96 (3.69, 4.24)	1.70 (1.64, 1.76)
	LAW	13.58 ± 0.62	7.90 (7.39, 8.42)	2.39 (2.28, 2.51)
Fever	Pre-LAW	4.60 ± 0.05	Ref.	Ref.
	Transition period	7.07 ± 0.13	2.47 (2.23, 2.72)	1.53 (1.47, 1.60)
	LAW	10.13 ± 0.53	5.53 (5.08, 5.99)	2.20 (2.09, 2.32)
Redness & swelling	Pre-LAW	1.33 ± 0.03	Ref.	Ref.
	Transition period	2.49 ± 0.08	1.16 (1.02, 1.31)	1.82 (1.74, 2.02)
	LAW	3.58 ± 0.32	2.25 (1.99, 2.54)	2.69 (2.45, 2.96)
Induration	Pre-LAW	0.76 ± 0.02	Ref.	Ref.
	Transition period	1.38 ± 0.06	0.62 (0.52, 0.74)	1.83 (1.65, 2.02)
	LAW	2.27 ± 0.25	1.51 (1.31, 1.74)	2.99 (2.66, 3.37)
Rare reaction	Pre-LAW	0.72 ± 0.02	Ref.	Ref.
	Transition period	0.98 ± 0.05	0.26 (0.17 0.36)	1.36 (1.22, 1.52)
	LAW	0.82 ± 0.15	0.10 (-0.03, 0.25)	1.14 (0.95, 1.35)

Pre-LAW stage refers to years between 2013 to the end of 2017. Transition period refers to years of 2018 and 2019. LAW stage targeted to 2020 since the vaccine administration law was came into force.

## Discussion

Reports from the Chinese Notifiable Diseases system reveal that the burden of viral hepatitis B is still high in China. Nearly seven percent of people in China are positive for HBsAg, that is, there are around 86 million hepatitis B virus carriers (17). Therefore, it is necessary to adhere to the strategy of HepB vaccination among residents especially children, to fundamentally control the spread of the disease (18, 19). Reports have shown that the coverage of hepatitis B vaccination for infants and children in China has been over 99% since 2015 (20). Assuring the safety of hepatitis B vaccines is one of the assurances that maintain high coverage (21).

This review focused on the safety of the most prevalent used HepB in China from 2013 to 2020. The annual number of HepB administrated doses in the mainland of China during the study period from 2013 to 2020 was used to calculate the rate of various AEFI concisely. We observed an increase in the rate of AEFI for HepB, which is in line with the general trend of AEFI reports by NASS and we believe is related to the general increase in the sensitivity of China’s AEFI monitoring system (11, 18). Compared to similar reports, we found lower rates of AEFI after HepB vaccination by our analysis (11, 22, 23). Since most of the rates of HepB AEFIs in previous studies were calculated by batch issuance data, the rate discrepancy reported in our review might be a result of the different denominator of the actual administrated dose (23). As vaccination sites usually stock vaccines over the required amount, there are delays and differences between the batch issuance data and the actual vaccination doses. Reports on the rates of AEFI based on issuance data showed that the surveillance sensitivity experienced a much slower increase around 15 per 100 thousand doses, compared with the rates from 3.1 to 14.8 per 100 thousand doses in the present analysis (24–26). However, the severe reaction rate was around one per million which is in line with the report by World Health Organization and similar reports in China (21).

Comparison between single and co-administration of HepB demonstrated a higher prevalence of Fever, Anaphylaxis Shock, Febrile Convulsion, and Lymphadenitis among those who received one or more extra vaccines at a time. These vaccines included other vaccines including DPT, Polio, and MenA/C.

Only one acute paralysis syndrome was reported in a case that received oral polio vaccine. However, all these severe reactions were at a very rare level and could not be defined as vaccine-induced reactions since they did not occur at a higher rate than the same events in children without HepB vaccination. The data presented in this report are only to generate hypotheses, because of the multiple limitations of passive surveillance data discussed above.

Improved sensitivity of the passive surveillance of AEFIs of HepB was found accompanying the legislative process of LAW in China, especially for common reactions like fever, redness, or induration. As we introduced before, AEFI surveillance before the issue of LAW legislation was guided and managed by several administrative orders including *Chinese Regulations on the Administration of Vaccine Circulation* and *Vaccination*, *Work Standard on Vaccination*, *Identification method for Adverse Reaction following Vaccination*, and *National Surveillance Guideline for Adverse Reaction following Immunization*. They were generally political reflections of vaccine events that happened during this period and also a result of increasing concern by the public on vaccine safety. Thus, we found a moderate rise in AEFI rates during the pre-LAW period from 2013 to 2017. With the gradual advancement of the legislative process of LAW, we found accelerating reported rates of AEFIs following HepB when the draft LAW was released for comments from the public. The LAW further defines the subjects of monitoring, investigation, diagnosis, treatment, analysis, and report, and puts forward new requirements such as adverse reaction management directory. And The introduction of supporting regulations within one year is required by LAW, which is the key to ensuring the implementation of the various systems stipulated in the Vaccine Administration LAW as soon as possible. Also, refresher training and sensitization of health care workers about reporting AEFI during the period of enactment of the Law was supposed to be conducted more frequently. The sensitivity then drastically increased to another platform when the LAW was officially coming into force at the end of 2019. From then on, the surveillance of AEFIs following vaccination was mandated by law, and the consciousness of safety reports might be also activated among the public and medical employees simultaneously. Similar situations could be found in other countries like the United States, when FDA Amendments Act was issued in 2007, adverse reports following immunization experienced a sustainable increase in the following years (27–29).

Besides, a spike in 2018 and subsequent decreases in 2019 and 2020 were found in the amount of AEFI records. This is suggested to be a result of joint effects of increased surveillance sensitivity of AEFI and fluctuating administrated HepB doses during these years (**Table 1**). According to the Chinese Statistical Yearbook, the birth rate in China has drastically decreased to 10.41‰ in 2019 and 8.52‰ in 2020. That explains why AEFI records decreased from 2019 to 2020, while the rates of AEFI experienced a stable increase. However, the rates of rare reactions only increased slightly but remained at a low level, reaffirming the safety of HepB even given the situation that surveillance sensitivity was much increased by LAW.

Fever has been the most frequently reported common reaction of HepB, followed by redness and swelling, and induration, which are suggested as part of the immune reaction of the body (30). The average rate was 3.1/100,000, which is in line with similar reports in China (31). Fever is common among infants below 1 year of age and is one of the AEFIs listed on the product label as being associated with HepB. However, studies have indicated that febrile response does not necessarily increase after HepB vaccination compared to the control group (32, 33). On the other hand, redness and swelling, and induration had been increasingly reported since 2015 probably due to the increase in surveillance sensitivity among adults (11, 23).

With respect to the severe adverse reactions to HepB, they have been reported only rarely worldwide (34). Nearly all of the rare reactions were reported in our data. Anaphylactic rash has been the most frequently reported. Studies have indicated the reason for this symptom is that the hepatitis B vaccine has not been fully shaken before injection, or the injection is shallow, which is a generalized phenomenon and causes the large particles contained in the vaccine to be absorbed slowly, resulting in the local reaction. Because erythema nodosum has also occurred after natural infection, such erythema may be an autoimmune reaction to HBsAg (35). In the meanwhile, Thrombocytopenic Purpura (TP) has been reported as another rare reaction with relatively more records. TP is thought to be caused by the presence of autoantibodies to glycoprotein IIb/IIIa molecules present in the platelet membrane (36). In infants whose idiotypic network is still forming, there is a higher likelihood of expression of cross-reactive autoantibodies after infection, immunization, or other environmental triggers (37).

Four cases of Arthus reaction have been detected during these eight years. Although Arthus reactions have been studied extensively in animals and have been reported to occur only rarely after immunization, these reactions have been reported to occur after skin testing with tetanus toxoid and after the administration of insulin (38). Arthus reactions are complement-dependent and neutrophil dependent. The formation of immune complexes by the meeting of antigens and antibodies in the vessel wall activates the classical Arthus reaction pathway (39). Thus, the strengthening dose of HepB followed by first immunization or the co-administration of other vaccines should get more attention for the prevention of Arthus reaction (34). However, studies have claimed that establishing a causal relationship between these diseases and HepB vaccination is difficult because these conditions are rare, have a poorly understood pathogenesis, occur in the absence of hepatitis B vaccination, and the onset of symptoms may be reported weeks to months after vaccination has occurred (40, 41).

There are several limitations to this review. There is no control group. Passive surveillance systems such as NASS are subject to limitations, including underreporting, unconfirmed diagnoses, and incomplete information in many reports. We could not deny the fact that active monitoring of adverse reactions could largely increase surveillance sensitivity by as much as two folds (42). However, with limited medical or social resources, it is still difficult to popularize active monitoring on a large scale at this moment. Passive surveillance like NASS is still the best and most feasible way to monitor AEFIs in China despite its limitation. Other limitations of NASS include its general inability to assess causality between an AEFI and the receipt of a vaccine. As the global health issue of COVID-19 started at the beginning of 2020, the SARS-CoV-2 vaccine and its safety brought much attention from the general population. Such influence on AE surveillance of HepB was not specified. As the highest rate of common AE was found in 2020, we could not specify whether such a situation is a result of the persistent influence of the LAW or a strengthened effect of COVID-19 or both. However, we found the highest rate of AEs of HepB in 2020, indicating a potential promoting effect on the AE surveillance of HepB by SARS-CoV-2 vaccine issue, as it also bears much public health significance. Moreover, we did not find a significant difference in the rates of rare reaction in 2020, which support the conclusion that rare reactions following HepB remained stable and rare.

## Conclusion

The legislation process of LAW largely increased the surveillance sensitivity of AEFIs of HepB but did not find new or unexpected AEFIs of concern associated with HepB among children aged below 12 months old. Most common AEFIs were local and systemic reactions including fever, redness, and induration. Severe reactions remained rare even though the surveillance sensitivity drastically increased. The report rates of serious abnormal reactions such as Anaphylactic shock, ARTHUS reaction, and Nervous system AEFI were all extremely rare. These data provide reliable evidence on the safety of HepB among infants in China. Guaranteed by law with the most strict and normal regulation, the LAW has largely increased the surveillance capability and sensitivity on AEFIs of HepB and also contributes to enhancing public confidence in HepB immunization. Further standardized AEFI investigation and assessment of causal association should be maintained. Continued assessment of the safety of HepB is needed as more children are vaccinated annually.

## Data availability statement

Original data are available on request. These were stored on password protected computers at Department of Laboratorial Science and Technology & Vaccine Research Center, School of Public Health, Peking University. Readers who wish to gain access to the data can write to the corresponding author. Requests to access these datasets should be directed to FC, cuifuq@bjmu.edu.cn.

## Ethics statement

The studies involving human participants were reviewed and approved by Peking University Institutional Review Board (IRB00001052-21008). Written informed consent from the participants’ legal guardian/next of kin was not required to participate in this study in accordance with the national legislation and the institutional requirements.

## Author contributions

CW, FC, SB, and XL conceived and designed the study, reviewed drafts of the paper, and approved the final draft. CW collected and analyzed the data, prepared figures, and tables, and authored drafts of the paper. SB, XL, and FC approved the final draft. QBL and NH collected the data and performed the investigations. CW, SB, and NH cleaned the data and prepared figures and tables. All authors have approved the final draft and agreed to the published version of the manuscript.

## Conflict of Interest

The authors declare that the research was conducted in the absence of any commercial or financial relationships that could be construed as a potential conflict of interest.

## Publisher’s note

All claims expressed in this article are solely those of the authors and do not necessarily represent those of their affiliated organizations, or those of the publisher, the editors and the reviewers. Any product that may be evaluated in this article, or claim that may be made by its manufacturer, is not guaranteed or endorsed by the publisher.
