# Supplementary material for: Change in adverse event reporting following immunization of hepatitis B vaccine among infants between 2013 to 2020 before and after the vaccine administration law in China
Source: Front Immunol. 2022 Sep 30;13:956473. doi: 10.3389/fimmu.2022.956473 (PMC9561938; doi:10.3389/fimmu.2022.956473)
Supplement: Supplementary file 1 [file Table_1.docx]

Table S1 Summary of the vaccine reactions by severity level

| Categories of AEFI | |  |  |
| --- | --- | --- | --- |
| Adverse reaction | Common, minor reaction | Fever | Classified into 3 categories:<37.0℃, 37.1℃ to 38.5℃, ≥ 38.6℃ |
|  |  | Redness & Swelling | Classified into 3 categories: none, 0.1cm to 2.5cm, ≥ 2.6cm |
|  |  | Induration | Classified into 3 categories: none, 0.1cm to 2.5cm, ≥ 2.6cm |
|  | Rare, serious reaction | Anaphylactic reaction | Anaphylactic rash |
|  |  |  | Thrombocytopenic Purpura |
|  |  |  | Angioedema |
|  |  |  | Allergic purpura |
|  |  |  | Anaphylaxis shock |
|  |  |  | Arthus reaction |
|  |  |  | vascular edema |
|  |  |  | Other anaphylactic reaction |
|  |  | Nervous system reaction | Vaccine Associated Paralytic Poliomyelitis |
|  |  |  | Acute disseminated encephalomyelitis |
|  |  |  | Guillain-Barre Syndrome |
|  |  |  | Encephalopathy |
|  |  |  | epilepsy |
|  |  |  | encephalitis and meningitis |
|  |  |  | multiple neuritis |
|  |  |  | brachial plexus neuritis |
|  |  |  | syncope |
|  |  |  | febrile convulsions |
|  |  |  | convulsions |
|  |  |  | Other nervous system reaction |
|  |  | Injection site reaction | Sterile abscess |
|  |  |  | Other injection site reaction |
|  |  | Bacillus Calmette-Guerin reaction | Disseminated BCG infection |
|  |  |  | Osteomyelitis |
|  |  |  | Local abscess |
|  |  |  | Lymphadenitis |
|  |  | Other rare reaction | Lymphitis/lymphadenitis |
|  |  |  | Others |
| Vaccine quality accident | | Damages to the tissues, organs and functions of the vaccine recipients, due to the unqualified quality of the vaccine in the process of vaccine production | |
| Vaccination accident | | Damages to the tissues, organs and functions of the vaccine recipients, due to the violation of administration guidance of vaccination, immunization procedures, vaccination program during the implementation of vaccination | |
| coincidence disease | | The recipient is in the incubation period or prodromal period of a certain disease at the time of vaccination, and the disease happens occasionally after vaccination. | |
| Psychogenic reaction | | Reactions due to psychological factors of the recipients during or after vaccination. | |

Table S2 National immunization program for child immunization procedure

| Vaccines | Abbreviation | Ages at vaccination | | | | | | | | | |
| --- | --- | --- | --- | --- | --- | --- | --- | --- | --- | --- | --- |
|  |  | Birth | 1^st^ month | 2^nd^ month | 3^rd^ month | 4^th^ month | 5^th^ month | 6^th^ month | 8^th^ month | 9^th^ month | 18^th^ month |
| Hepatitis B vaccine | HepB | 1 | 2 |  |  |  |  | 3 |  |  |  |
| Bacille Calmette-Guérin vaccine | BCG | 1 |  |  |  |  |  |  |  |  |  |
| Inactivated polio vaccine | IPV |  |  | 1 | 2 |  |  |  |  |  |  |
| Diphtheria, pertussis and tetanus vaccine | DTP |  |  |  | 1 | 2 | 3 |  |  |  | 4 |
| Measles mumps rubella vaccine | MMR |  |  |  |  |  |  |  | 1 |  | 2 |
| Live attenuated and activated Japanese encephalitis vaccine | JE-L |  |  |  |  |  |  |  | 1 |  |  |
| Japanese Encephalitis Inactivated vaccine | JE-I |  |  |  |  |  |  |  | 1,2 |  |  |
| Group A meningococcal polysaccharide vaccine | Men A |  |  |  |  |  |  | 1 |  | 2 |  |
| Group A and group C meningococcal polysaccharide vaccine | Men A/C |  |  |  |  |  |  | 1 |  | 2 |  |
| Live Hepatitis A vaccine | HepA-L |  |  |  |  |  |  |  |  |  | 1 |
| Inactivated Hepatitis A vaccine | HepA-I |  |  |  |  |  |  |  |  |  | 1 |

Table S3 Prevalence of common and rare reactions between single and co-administration of HepB.

| Reactions | | Single Hep-B | |  | 1 or more other vaccines | |
| --- | --- | --- | --- | --- | --- | --- |
|  |  | n | % |  | n | % |
| Fever | Normal | 3591 | 69.90 |  | 1549 | 30.10 |
|  | (37.1 to 38.5)℃ | 2498 | 46.20 |  | 2909 | 53.80 |
|  | ≥ 38.6℃ | 1857 | 36.40 |  | 3238 | 63.60 |
| Redness and swelling | Normal | 5556 | 45.50 |  | 6662 | 54.50 |
|  | (0.1 to 2.5)cm | 1222 | 70.40 |  | 515 | 29.60 |
|  | 2.6cm to 5cm | 1014 | 70.10 |  | 433 | 29.90 |
|  | ≥5cm | 154 | 64.20 |  | 86 | 35.80 |
| Induration | Normal | 6518 | 47.70 |  | 7153 | 52.30 |
|  | (0.1 to 2.5)cm | 837 | 73.60 |  | 301 | 26.40 |
|  | 2.6cm to 5cm | 542 | 71.30 |  | 218 | 28.70 |
|  | ≥5cm | 49 | 67.10 |  | 24 | 32.90 |
| Thrombocytopenic Purpura |  | 36 | 66.7 |  | 18 | 33.3 |
| Anaphylactic reaction | Anaphylactic rash | 699 | 59.4 |  | 478 | 40.6 |
|  | Angioedema | 9 | 60.00 |  | 6 | 40.00 |
|  | Allergic purpura | 7 | 70.00 |  | 3 | 30.00 |
|  | Anaphylaxis shock | 3 | 42.90 |  | 4 | 57.10 |
|  | Arthus reaction | 3 | 75.00 |  | 1 | 25.00 |
|  | Other anaphylactic reaction | 30 | 61.2 |  | 19 | 38.8 |
| Nervous system reaction | Febrile convulsion | 2 | 14.3 |  | 12 | 85.7 |
|  | Convulsion/Seizures | 4 | 66.7 |  | 2 | 33.3 |
|  | Encephalopathy | 2 | 66.7 |  | 1 | 33.3 |
|  | Acute paralysis syndrome | 0 | 0 |  | 1 | 100 |
| Injection site reaction | Sterile abscess | 43 | 79.6 |  | 11 | 20.4 |
| Lymphitis/lymphadenitis |  | 1 | 20.0 |  | 4 | 80.0 |
| Other rare reaction |  | 14 | 43.8 |  | 18 | 56.2 |

Table S4 Rates of Common reaction following Hep-B immunization by years (per 100,000 vaccinated doses)

| Common reaction | | Total | | 2013 | | 2014 | | 2015 | | 2016 | | 2017 | | 2018 | | 2019 | | 2020 | |
| --- | --- | --- | --- | --- | --- | --- | --- | --- | --- | --- | --- | --- | --- | --- | --- | --- | --- | --- | --- |
|  |  | Cases | Rates | Cases | Rates | Cases | Rates | Cases | Rates | Cases | Rates | Cases | Rates | Cases | Rates | Cases | Rates | Cases | Rates |
| Fever | 37.1 to 38.5℃ | 5407 | 3.1 | 117 | 0.7 | 296 | 1.9 | 437 | 2.1 | 604 | 2.9 | 657 | 3.2 | 1164 | 3.3 | 959 | 4.0 | 1173 | 6.2 |
|  | ≥ 38.6℃ | 5095 | 2.9 | 222 | 1.3 | 407 | 2.6 | 423 | 2.1 | 585 | 2.8 | 623 | 3.0 | 1144 | 3.2 | 955 | 4.0 | 736 | 3.9 |
| Redness & swelling | 0.1 to 2.5cm | 1737 | 1.0 | 23 | 0.1 | 83 | 0.5 | 122 | 0.6 | 175 | 0.8 | 220 | 1.1 | 378 | 1.1 | 321 | 1.3 | 415 | 2.2 |
|  | 2.6cm to 5cm | 1447 | 0.8 | 41 | 0.2 | 68 | 0.4 | 98 | 0.5 | 149 | 0.7 | 183 | 0.9 | 405 | 1.1 | 280 | 1.2 | 223 | 1.2 |
|  | ≥5cm | 240 | 0.1 | 10 | <0.1 | 33 | 0.2 | 19 | <0.1 | 14 | <0.1 | 25 | 0.1 | 63 | 0.2 | 39 | 0.2 | 37 | 0.2 |
| Induration | 0.1 to 2.5cm | 1138 | 0.7 | 18 | 0.1 | 69 | 0.4 | 82 | 0.4 | 121 | 0.6 | 141 | 0.7 | 256 | 0.7 | 161 | 0.6 | 290 | 1.5 |
|  | 2.6cm to 5cm | 760 | 0.4 | 17 | 0.1 | 43 | 0.3 | 52 | 0.3 | 60 | 0.3 | 78 | 0.4 | 177 | 0.5 | 211 | 0.9 | 122 | 0.7 |
|  | ≥5cm | 73 | <0.1 | 8 | <0.1 | 14 | <0.1 | 5 | <0.1 | 7 | <0.1 | 4 | <0.1 | 11 | <0.1 | 9 | <0.1 | 15 | <0.1 |

Table S5 Rates of total clinical diagnosis following Hep-B immunization by years (per 1 million vaccinated doses).

| **Diagnosis** | Total | | | 2013 | | | 2014 | | | 2015 | | | 2016 | | | 2017 | | | 2018 | | | 2019 | | | 2020 | | |
| --- | --- | --- | --- | --- | --- | --- | --- | --- | --- | --- | --- | --- | --- | --- | --- | --- | --- | --- | --- | --- | --- | --- | --- | --- | --- | --- | --- |
|  | Cases | | Rate | Cases | | Rate | Cases | | Rate | Cases | | Rate | Cases | | Rate | Cases | | Rate | Cases | | Rate | Cases | | Rate | Cases | | Rate |
| **Total** | 1431 | 8.2 | | 97 | 5.6 | | 160 | 10.2 | | 102 | 5.0 | | 174 | 8.4 | | 155 | 7.5 | | 323 | 9.0 | | 265 | 11.1 | | 155 | 8.2 | |
| **Anaphylactic reaction** |  |  | |  |  | |  |  | |  |  | |  |  | |  |  | |  |  | |  |  | |  |  | |
| Anaphylactic rash | 1177 | 6.8 | | 80 | 4.6 | | 139 | 8.8 | | 81 | 4.0 | | 146 | 7.0 | | 122 | 5.9 | | 262 | 7.3 | | 218 | 9.1 | | 129 | 6.8 | |
| Thrombocytopenic (Purpura) | 54 | 0.3 | | 1 | <0.1 | | 2 | 0.1 | | 6 | 0.3 | | 3 | 0.1 | | 6 | 0.3 | | 20 | 0.6 | | 10 | 0.4 | | 6 | 0.3 | |
| Angioedema | 15 | <0.1 | | 1 | <0.1 | | 3 | 0.2 | | 2 | <0.1 | | 1 | <0.1 | | 0 | 0 | | 4 | 0.1 | | 3 | 0.1 | | 1 | <0.1 | |
| Allergic purpura | 10 | <0.1 | | 1 | <0.1 | | 1 | <0.1 | | 1 | <0.1 | | 1 | <0.1 | | 3 | 0.1 | | 0 | 0 | | 1 | <0.1 | | 2 | 0.1 | |
| Anaphylaxis shock | 7 | <0.1 | | 1 | <0.1 | | 1 | <0.1 | | 0 | 0 | | 0 | 0 | | 2 | <0.1 | | 2 | <0.1 | | 1 | <0.1 | | 0 | 0 | |
| Arthus reaction | 4 | <0.1 | | 0 | 0 | | 3 | 0.2 | | 0 | 0 | | 0 | 0 | | 1 | <0.1 | | 0 | 0 | | 0 | 0 | | 0 | 0 | |
| Other anaphylactic reaction | 49 | 0.3 | | 1 | <0.1 | | 1 | <0.1 | | 9 | 0.4 | | 5 | 0.2 | | 4 | 0.2 | | 15 | 0.4 | | 13 | 0.5 | | 1 | <0.1 | |
| **Nervous system reaction** |  |  | |  |  | |  |  | |  |  | |  |  | |  |  | |  |  | |  |  | |  |  | |
| Febrile convulsion | 14 | <0.1 | | 2 | 0.1 | | 1 | <0.1 | | 0 | 0 | | 1 | <0.1 | | 3 | 0.1 | | 2 | <0.1 | | 2 | <0.1 | | 3 | 0.2 | |
| Convulsion/Seizures | 6 | <0.1 | | 2 | 0.1 | | 0 | 0 | | 0 | 0 | | 2 | <0.1 | | 1 | <0.1 | | 0 | 0 | | 0 | 0 | | 1 | <0.1 | |
| Encephalopathy | 3 | <0.1 | | 0 | 0 | | 0 | 0 | | 0 | 0 | | 0 | 0 | | 0 | 0 | | 2 | <0.1 | | 1 | <0.1 | | 0 | 0 | |
| Acute paralysis syndrome | 1 | <0.1 | | 0 | 0 | | 0 | 0 | | 0 | 0 | | 0 | 0 | | 0 | 0 | | 0 | 0 | | 1 | <0.1 | | 0 | 0 | |
| **Sterile abscess** | 54 | 0.3 | | 4 | 0.2 | | 8 | 0.5 | | 2 | <0.1 | | 5 | 0.2 | | 9 | 0.4 | | 10 | 0.3 | | 9 | 0.4 | | 7 | 0.4 | |
| **Lymphitis/lymphadenitis** | 5 | <0.1 | | 2 | 0.1 | | 0 | 0 | | 0 | 0 | | 1 | <0.1 | | 1 | <0.1 | | 0 | 0 | | 0 | 0 | | 1 | <0.1 | |
| **Other rare reaction** | 32 | 0.2 | | 2 | 0.1 | | 1 | <0.1 | | 1 | <0.1 | | 9 | 0.4 | | 3 | 0.1 | | 6 | 0.2 | | 6 | 0.3 | | 4 | 0.2 | |
